# Augmented Reality (AR) for Surgical Robotic and Autonomous Systems: State of the Art, Challenges, and Solutions

**DOI:** 10.3390/s23136202

**Published:** 2023-07-06

**Authors:** Jenna Seetohul, Mahmood Shafiee, Konstantinos Sirlantzis

**Affiliations:** 1 Mechanical Engineering Group, School of Engineering, University of Kent, Canterbury CT2 7NT, UK; 2 School of Mechanical Engineering Sciences, University of Surrey, Guildford GU2 7XH, UK; 3 School of Engineering, Technology and Design, Canterbury Christ Church University, Canterbury CT1 1QU, UK; konstantinos.sirlantzis@canterbury.ac.uk; 4 Intelligent Interactions Group, School of Engineering, University of Kent, Canterbury CT2 7NT, UK

**Keywords:** augmented reality (AR), machine learning (ML), navigation, planning, robotic and autonomous systems (RAS), surgery

## Abstract

Despite the substantial progress achieved in the development and integration of augmented reality (AR) in surgical robotic and autonomous systems (RAS), the center of focus in most devices remains on improving end-effector dexterity and precision, as well as improved access to minimally invasive surgeries. This paper aims to provide a systematic review of different types of state-of-the-art surgical robotic platforms while identifying areas for technological improvement. We associate specific control features, such as haptic feedback, sensory stimuli, and human–robot collaboration, with AR technology to perform complex surgical interventions for increased user perception of the augmented world. Current researchers in the field have, for long, faced innumerable issues with low accuracy in tool placement around complex trajectories, pose estimation, and difficulty in depth perception during two-dimensional medical imaging. A number of robots described in this review, such as Novarad and SpineAssist, are analyzed in terms of their hardware features, computer vision systems (such as deep learning algorithms), and the clinical relevance of the literature. We attempt to outline the shortcomings in current optimization algorithms for surgical robots (such as YOLO and LTSM) whilst providing mitigating solutions to internal tool-to-organ collision detection and image reconstruction. The accuracy of results in robot end-effector collisions and reduced occlusion remain promising within the scope of our research, validating the propositions made for the surgical clearance of ever-expanding AR technology in the future.

## 1. Introduction

Over the past couple of decades, significant advancements in the performance of robotic platforms have been achieved by researchers in the academic community, with the deployment of such robots soaring amidst the COVID-19 pandemic. Studies show that the high probability of a resurgence in COVID-19 cases necessitates cost-effective and self-deploying telepresence robots to ensure pathogen control worldwide [1]. According to Raje et al. [2], the market size of healthcare robots was over 9 billion in 2022, exceeding the current fleet number more than twofold in comparison to values in 2019. Today, robotic platforms such as the Davinci robot have significantly improved the way in which surgeons perform complex interventions, reducing the need for patient re-admission due to its minimally invasive nature. Novel surgical robots are, today, the most sought-after approach in performing repetitive tasks in an accurate manner. Imaging technology has significantly changed the world of robotic surgery, especially when it comes to biopsies, the examination of complex vasculature for catheterization, and the visual estimation of target points for port placement. There is a great need for the image analysis of CT scans and X-rays for the identification of the correct position of an anatomical landmark such as a tumor or polyp. This information is at the core of most augmented reality systems, where development starts with the reconstruction and localization of targets. Hence, the primary role of augmented reality (AR) applications in surgery would be to visualize and guide a user towards a desired robot configuration with the help of intelligent computer vision algorithms.

The application of such cutting-edge robotic technologies remains diverse in various sectors, from carrying out military or manufacturing tasks to airborne or underwater operations, due to their dexterity, ease of operation, high adaptability, and multi-functionality. The widespread demand for AR in surgery is the impetus for our work, with a core focus on the challenges encountered in their deployment in the existing literature as well as our proposed solutions in counteracting these issues, emphasizing the precision of end-effector placement and feedback from control systems. The field of surgery has seen a quantum leap in the evolution of procedural ergonomics and the level of autonomy in robots during an intervention. Since the first robotic-assisted surgery was successfully used to treat neurological tumors from a spin-out industrial robot called the PUMA 200 in 1985 [3], scientists across the globe have found the need for increased precision in robotic arm positioning and orientation to relieve surgeons of their long hours in operation theaters. From the AESOP robotic arm built by Computer Motion for laparoscopic camera positioning in 1993 to the DaVinci system cleared for use in 2000 for countless segmentectomies of various organs, each platform has been improved in terms of hardware and software features, introducing the 3D visualization of the inner anatomy to surgeons via a see-through display. From this evolutionary hierarchy, scientists have seen the use of AR as a blessing in the surgical setting, reducing the surgeon’s cognitive load whilst performing complex surgeries such as cardiothoracic, colorectal, head and neck, and urological resections.

The collaboration between AR and RAS is a breakthrough in the world of minimally invasive robotic surgeries, with the earliest publications on this principle dating back to the 2000s, by Worn et al. [4]. More recently, in the media, the healthcare startup company Novarad introduced an AR-based surgical navigation system called VisAR, which operates based on virtual organ superposition with submillimeter accuracy [5]. Various other startups, such as Proximie [6], have also emphasized the importance of AR for surgical guidance through their extensive work on virtual “scrubbing in” and the augmented visualization of end-effector operations. These platforms provide an incentive to surgical robot manufacturers to integrate similar collaborative software packages into their control systems to obviate the risk of hand tremors, improving the synergy in human–robot arm placement and enabling telepresence via microphone communication throughout the procedure. This type of collaboration remains in its early pilot stages, although it is of increasing relevance in addressing the gaps in non-contact surgery during the pre- and post-pandemic eras.

Although most advanced robots perform pre-programmed repetitive tasks with minimal artificial intelligence (AI) training, existing surgical robots with supplementary visual, olfactory, and haptic modalities prove to augment human–robot interaction and hence improve overall system performance. In this paper, we evaluate the types of surgical scenarios that involve AR technology, their corresponding navigation strategies, and the DL methods used in their operation. We also focus on identifying the loopholes in the existing literature, involving the levels of autonomy (LoA) in surgical RAS, accuracy in GPU performance, experiment genericity, and clinical evaluation, amongst others. In the conventional robot hierarchy adapted from works by Haidegger et al. [7] and Attanasio et al. [8], the level of autonomy (LoA) framework enables researchers to adapt the control system to terrain irregularities and exploit the force requirements for more efficient robot kinematics. Using such robot classification, researchers across the academic community have explored the possibilities of enhancing the control systems of surgical robots with novel visual modalities, for better output efficiency within the accepted medical standards. Lower-level robots (0–1) are employed for assigned tasks within a defined scope, requiring a surgeon’s guidance and pre-programming with no or limited external support, such as active constraints for user navigation or virtual fixtures to improve user visualization of the surrounding anatomy. In the higher-level entities (2–5), more comprehensive systems have been developed for varying surgical complexity, which are environment-aware and perform cognitive decision making whilst adapting to external changes in stimuli. Such systems can provide certain capabilities to the human–robot interface to relieve the surgeon of certain responsibilities whilst switching from operator to robot for the duration of the task to be executed. To some degree, it must be noted that algorithmic approaches are included in each LoA (Level 0 has a degree of tremor filtering and redundancy resolution) but mostly the higher-level platforms are able to perform preoperative planning and devise an interventional algorithm to allow complete control of the surgery under a surgeon’s supervision.

The ability to perform surgeries autonomously has been debated by several law courts against Intuitive Surgical, with up to 3000 cases in 2016 [9]. This is because certain surgical robots require a degree of human input in line with the medical equipment safety design concept, which includes the Medical Electrical Equipment Standard (IEC 80601-2-77 [10] (https://www.iso.org/standard/68473.html) [accessed on 20 March 2023] as well as the IEC60601-1 [11] (https://www.iso.org/standard/65529.html) [accessed on 20 March 2023] safety standard series [9]. The latter describes the standards that manufacturers must follow to ensure patient (target) and surgical team safety (surgeon as main user), incorporating risk control measures while controlling the robot in a surgery. Furthermore, the patient safety guidance under the standards states that, owing to the vulnerability of patients, the surgical team needs to be well-versed in hazard and risk prevention in case of an accident. For example, any uninitiated motion or swerving away from the trajectory, excessive speed in the motors, or faulty safeguards may pose life-threatening risks during a pre-planned autonomous or semi-autonomous thoracotomy through 3D image overlay. Our review does not include fully autonomous robotic platforms that can operate without human intervention and employ sensory feedback systems for a decentralized network, due to the high risks associated with complete robot autonomy. Instead, we classify and identify the features within the exhaustive list of surgical robotic platforms, adapted from works by Simaan et al. [12] and Hoeckelmann et al. [13], which are or have the potential of providing a certain LoA to the surgeon through visualization methods or other DL algorithmic approaches. These robots are either on the market currently as commercial robots or proof-of-concept devices that have deployable potential in the future.

### 1.1. Current Knowledge of XR, AR, and VR Platforms

The umbrella term for platforms used for immersive visualization, interaction, simulation, and improved user perception of scenarios is called extended reality (XR). AR systems have proven to be an indispensable medium for human interaction with the external virtual world by bridging the gap between the required task and assisting tools through unobscured user display. According to Krevelen et al. [14], AR provides user immersion by augmenting the field of view of the real world with computerized data such as graphics and audiovisual content, as well as other sensory reinforcement methods. Several interfaces have been used in robots, such as head-mounted displays, smart glasses such as the HoloLens 1 and 2 [15], and handheld devices such as smartphones and overhead projectors [16]. The HoloLens is likened to a personal computing system, designed with an optical see-through mechanism: virtual data are projected onto a translucent screen in the user’s field of view (FoV) while preserving the real-world setting in the background; this enables the instant synchronization of proprioceptive stimuli, as well as complete situational awareness. This allows the device to fit into different sectors, such as gaming, manufacturing, and surgery, due to its high-resolution imaging, albeit with sub-optimal spatial coherence. It is fitted with tracking sensors, pose estimation sensors, 3D coordinate mapping sensors, environment-sensing cameras, speakers, inertial measurement units (IMU), and holographic processing units. In other words, an AR model for surgery is a revolutionary platform aiming to create and display digital information in real time, primarily superposed over the actual organ. The three main components of this model include a physical object such as forceps or grippers, used as a prototype for the virtual design and interpretation; ML algorithm-driven sensors with cameras for the visual depiction of output images; and modeling software that processes the input signals from the cameras [17].

On the other hand, virtual reality (VR) creates a computer-generated back-end scene for complete immersion, such that the user can experience real-world scenes in a completely virtual environment. The supporting device system for the generation of a virtual world consists of joysticks, controllers, or trackpads for navigation; head-tracking devices for pose estimation; and microphones for voice recognition. VR headsets such as the Oculus Rift (Facebook, Menlo Park, CA, USA) [18] and Oculus Quest [19] tend to blur the user’s real-life environment and create a completely immersive virtual scenario, controlled by a stereoscopic binocular system. The virtual scenario is then developed for the user by projecting different 2D images onto each eye, with varying perspectives and fields of view (FoV) between 90° and 210° and a speed of 90 frames per second, for an enhanced immersive experience [20]. VR pose estimation in surgical settings includes the use of clinically acquired imaging datasets, which are reconstructed in a dexterous workspace with geometrical x, y, and z planes. This enables motion tracking using fiducial cues registered onto specific coordinate planes that have been isolated from a reconstructed virtual scenario and replaced in the exact positions after removing the back-end background [21].

### 1.2. Definition and Scope of Augmented Reality in Surgery

Since its introduction to the scientific world in the 2000s, AR in surgery has been developing at a soaring rate, although it has been criticized by many due to the heavy wearable devices, limited sensory input, and inefficient real-time object registration due to tissue deformation [22]. The new era of AR in RAS has seen a leap in computer-vision-based decision making, as stated in a paper by Nilsson et al. [23], hence proving its efficacy in fields including, but not limited to, machinery, manufacturing, surgery, and education. Following Halsted’s approach of training, “see one, do one, teach one”, a scientist may observe a particular task being performed through an augmented visualization device, practice this task several times until mastery is achieved, and eventually demonstrate this concept to trainees [24]. To provide an accurate representation of the role of AR, we compile and examine the definitions stated by Milgram et al. [25] and Azuma et al. [26], who claimed that AR is defined as “the augmentation of natural feedback such as visual, haptic and olfactory feedback to the surgeon using fiducial cues”. We decided to follow this definition and classify the existing literature papers in our meta-analysis according to this principle, excluding VR and AV papers, as well as the side-by-side visualization of medical images during a surgical procedure without superposition or virtual-to-real tool alignment.

Despite the prevalence of AR technologies in several sectors, there is a significant gap in their performance in handling cross-modalities during surgical manipulation, which may lead to targeting errors and inaccuracies such as false negatives. This paper aims to conduct a systematic review of different types of state-of-the-art surgical robotic platforms while identifying areas for technological improvement. We associate specific control features, such as haptic feedback, sensory stimuli, and human–robot collaboration, with AR technology to perform complex surgical interventions for increased user perception of the augmented world. Current researchers in the field have, for long, faced innumerable issues with low accuracy in tool placement around complex trajectories, pose estimation, and difficulty in depth perception during two-dimensional medical imaging. The plethora of robots described in this review, such as Novarad and SpineAssist, are analyzed in terms of their hardware features, computer vision systems (such as deep learning algorithms), and the clinical relevance of the literature. We attempt to outline the shortcomings in current optimization algorithms for surgical robots (such as YOLO and LTSM) whilst providing mitigating solutions to internal tool-to-organ collision detection and image reconstruction. Our paper presents a stepping stone for researchers to explore the possibilities of adapting AR to RAS for the navigation and control of surgical tools within a plethora of anatomical environments.

The organization of this paper is as follows. Section 2 presents the data collected during the literature review, including the commercial robotic platforms, proof-of-concept systems, their operating principles, and the corresponding AR human–robot interfaces. Section 3 outlines the working principles of AR devices and the categories of hardware devices used in line with the AR systems for accurate visualization. Section 4 emphasizes the software implementation in these AR models, as well as the corresponding input and output data obtained. Section 5 introduces the novel DL framework used for object detection, path planning, and the data analysis of medical image datasets. Section 6 opens the floor to a discussion about the various challenges faced by the robot platform whilst interfacing with AR technology, such as risks of collisions, reduced collaboration, and divergence in trajectories, as well as some solutions to combat these issues. Section 6 provides a summary of the paper while addressing future research possibilities in AR for surgery. Finally, a concluding statement in Section 7 provides an incentive to surgeons and researchers to elaborate and improve the given solutions in this discussion.

## 2. Research Background

### 2.1. Classification of AR–RAS Collaboration in Meta-Analysis Study

**Figure 1:**
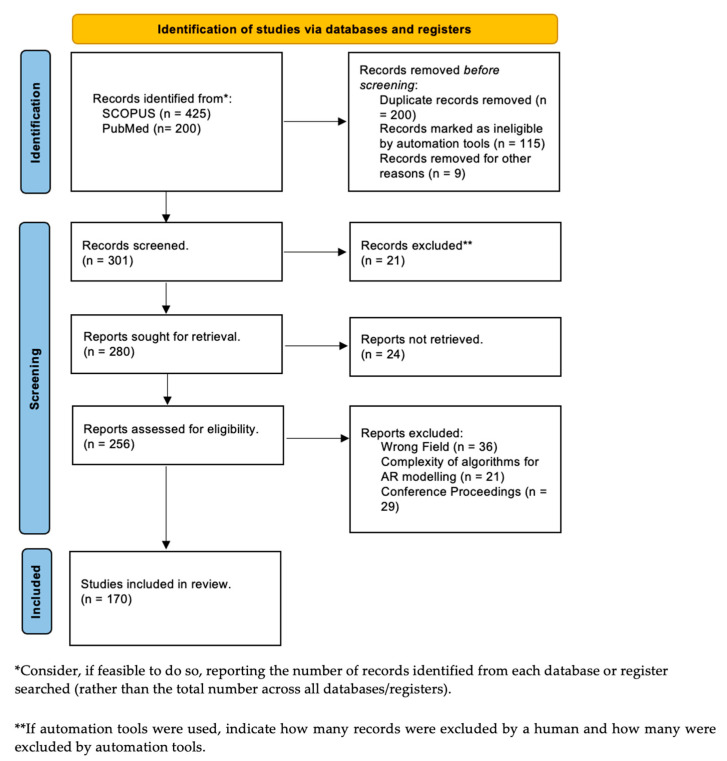
Systematic review results in PRISMA flowchart format, identifying the duplicates and excluded papers.

**Figure 2:**
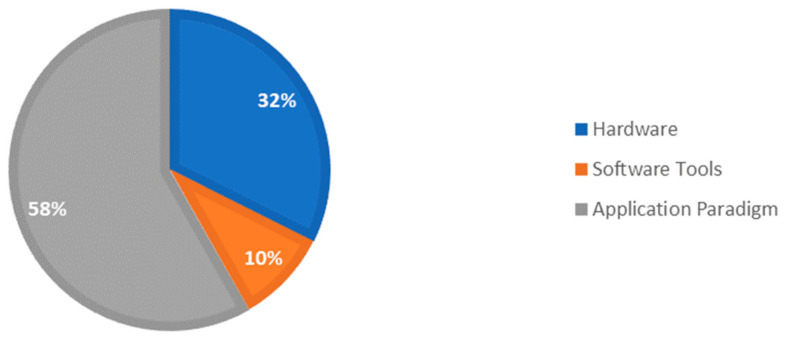
Pie chart showing the distribution and taxonomy of retrieved papers from literature.

We started our search with papers obtained from several peer-reviewed databases, such as IEEExplore, Google Scholar, SCOPUS, and PubMed, to perform a thorough initial literature search (Figure 1). We focused on articles published from the last decade till March 2023, due to the rapid advancement of AR technologies, which was marked by the groundbreaking release of the Microsoft HoloLens in 2016 [27]. The key search terms used to triage the papers from these databases were from the title and the abstract, such as “Augmented Reality” AND “Robots” AND “Surgery” OR “Surgical Robot” OR “Surgical Robotics” OR “Surgical Navigation” OR “Robot Assisted” OR “Minimally Invasive Surgery”. Ultimately, the methodological segregation of papers was performed, dividing them into clusters in line with the ATRA framework in [28]. They were then divided into different groups, such as “Software Tools for Surgical Applications”, “Hardware Specifications”, and “DL Algorithms” (see Figure 2), which led to a total of 425 papers from SCOPUS and 200 papers from PubMed, excluding articles that were not relevant to our review, duplicated, or published in non-English languages. Considering the fact that the papers reviewed were published over the last 20 years, there was a significant gap in the literature regarding high-level AR and AI applications for robots in surgery. The number of papers published on surgical robots based on AR before the year 2013 was less than 20 per year, and the accrued number of papers published was lower than 500. The literature review conducted in 2023 saw the highest increase in papers. The techniques listed above underwent a detailed review amongst the wealth of peer-reviewed publications, whereby the advantages and disadvantages of the available robotic systems, their AR-based control features, and the computational algorithms were analyzed. Parameters such as 3D image reconstruction, types of hardware features, and the potential gaps found in clinical evaluation and path planning were reviewed. Some authors focused on image-guided control and navigation using intelligent predictive systems, while others studied the orientation and positioning of robots in an augmented view, from point cloud mesh generation to various algorithmic approaches.

### 2.2. Review of Commercial Robots and Proof-of-Concept Systems

The focus of our paper remains the increasing demand for visualization in surgical robotics, especially in preoperative scenarios, onto the real-world patient from the object detection process of landmarks of interest beyond the visible surface and by merging preoperative and real-world images together. A higher rate of deployment of AR devices in surgery is encouraged more than ever today, stemming from the expected increase in the market demand of at least 18.02% by the end of 2023. The most common areas of robotic surgery employing AR at present include neurosurgery, cardiothoracic, orthopedic, gastrointestinal, and ENT, amongst others [29]. For example, a surgeon may use the preoperative imaging from a patient’s medical database to locate a cancerous tumor and project this reconstruction onto the real anatomy to help them to find its exact position. For further reading on the types of robotic surgeries performed, readers may refer to Robotic Assisted Minimally Invasive Surgery: A Comprehensive Textbook [30]. Expanding on the works by Barcali et al. [31], Appendix A classifies the types of commercial robots and proof-of-concept systems that we concentrate on in this paper in terms of the parameters studied, AR interfaces, anatomical location, and CE marking awarded.

In this paper, we aim to contribute to future research by building a foundation on the current state of the art and proof of concept in AR for surgical robotics, whilst addressing the following research questions:

- What is the current state-of-the-art research in integrating AR technologies with surgical robotics?
- What are the various hardware and software components used in the development of AR-assisted surgical robots and how are they intertwined?
- What are some of the current application paradigms that have enhanced these robotic platforms? How can we solve the research gaps in previous literature reviews and promote faster performance and accuracy in image reconstruction and encourage high LoA surgical robots with computer vision methods?

**Figure 3:**
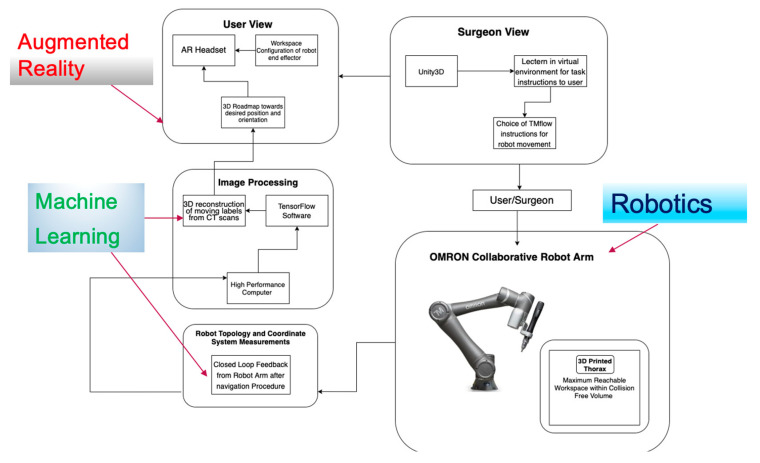
Logic relationship between the different sections evaluated in the literature review, such as hardware (robotic platforms), software (machine learning algorithms and calibration technologies), and augmented reality headsets.

To understand and elaborate on the methodologies used in AR-based robotic surgeries, we decided to classify the systems in terms of their hardware and software features as an initial literature search from our meta-analysis and based on the logic relationship in Figure 3. This section focuses on the features of the AR interfaces in RAS that contribute to the hardware development of the system. The papers are categorized in terms of their different marker trackers and sensors, their image registration and alignment methods, and the types of displays used for visualization.

## 3. Hardware Components

### 3.1. Patient-to-Image Registration Devices

In surgical navigation systems, AR-based scenes require seamless interaction between the real world and the digital world, increasing the need for precise motion tracking via marker-based or location-based mechanisms [32]. Often, pose estimation via motion tracking enables the user to perform the accurate manipulation of tools and geometrically position end-effectors for the cutting, outlining, and extraction of anatomical landmarks such as shoulder blades or internal organs. Location-based triggers may be used in conjunction with, but not limited to, pose estimation sensors such as IMUs, which provide several measurements, such as acceleration, magnetic field strength, and orientation angles. There is also the possibility of obtaining accurate geographical locations of specific clinical personnel through AR screens such as smartphones, HMDs, and even smart glasses [33]. These markers provide a basis for the initial alignment of the virtual world to the real world, with respect to a generic reference frame in space towards the target of interest.

**Figure 4:**
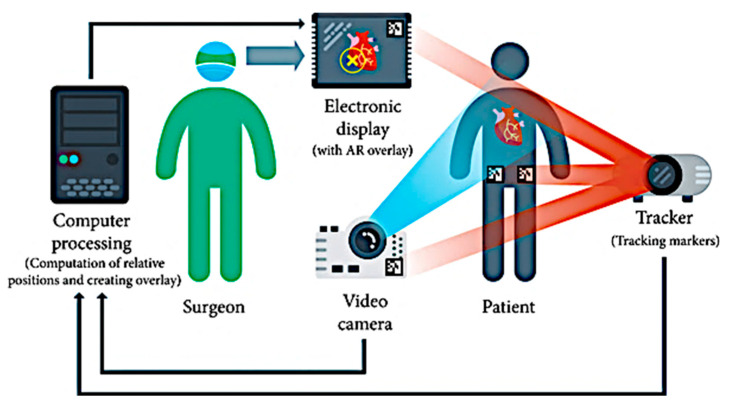
The tracking process during AR alignment between patient and device.

Contrary to marker-based AR calibration systems, which use pre-defined tracking markers such as QR codes to leverage objects onto a real-world scene, markerless systems tend to enable user-friendly referencing cues to position an object in space. They operate by experimenting with different human skin textures, internal vessel structures, and geometrical features from medical scans of a patient [34]. The user can prescribe the location of the model and navigate around the scene without necessarily disturbing the external aspects of their surroundings, collating relayed data from accelerometers and visual, haptic, and olfactory sensors, as well as GPS systems. Such AR models depend on computer vision algorithms such as convolutional neural networks (CNN) to perceive target objects without fiducial markers, commonly trained using a software program called TensorFlow API. The specific referencing points are passed through such neural networks in real time, such that the accurate positions of the user can be tested and validated in further experimental procedures (see Figure 4).

There exist a multitude of sensors that are integrated into robotic platforms for the detection of precise locations in a surgical procedure, ranging from ultrasonic sensors [35], mechanical sensors [36], and electromagnetic (EM) sensors [36] to optical tracking sensors [37]. Today, the most acclaimed sensors for image-guided surgical navigation systems include optical and EM tracking sensors. AR display systems require an integrated camera tracking mechanism, which involves the registration of the head location and direction. This process can be performed using different individual or a combination of tracking sensors, with a wide range of applications in the clinical sector, e.g., devices such as Polaris and MiniBird (Ascension Technology Corp., Milton, VT, USA), which attach to the surgeon’s head for accurate simultaneous localization and mapping (SLAM). This is the process by which a surgical robotic tool can construct and generate a collision-free map and simultaneously identify its exact location using the map. It uses different filtering techniques, such as Kalman filters (KF), particle filters, and graph-based SLAM. A range of ML algorithms [38] are used in the development of a navigation structure in a discrete-time state-space framework, such as the unscented KF, which approximates the state distribution with a Gaussian Random Variable, where the posterior mean and covariance are captured for propagation through the nonlinear system; the extended KF, to overcome the linearity assumption for the next probability state; and the Monte Carlo sequential algorithms for filtering through the estimation of trajectory samples. Other graphical SLAM techniques adopt a node-to-node graph formulation technique, where the back end enables robot pose correction in order to produce an independent topology of the robot, as explained in [39]. The most common SLAM algorithm used in surgery includes the visual SLAM, based on monocular and trinocular RGB-D camera images that use information from the surrounding surgical environment to track the 3D landmarks through Bayesian methods, as cited in the literature [40]. In this section, we focus on the surgery SLAM applications, where several examples of surgical tracking systems are given, typical of robotic platforms with AR integration: specialized robot manipulators for surgery, control algorithms for AR visualization, and ML decision-making algorithms, amongst others.

(i) Electromagnetic Tracking Systems (EMTs)

Contrary to mechanical tracking, which depends on the positions of end-effectors to deliver fast update rates via rotary encoders or potentiometers, electromagnetic (EM) tracking uses a stationary source with orthogonal magnetic coils within an operating range of 1–3 m. Nowadays, the only AR technique that limits conventional occlusion limits is EM tracking, which operates based on field generation placed near the patient and connected to the latter by coil wires [41]. The orientation and position of the tracking sensors are based on the signal attenuation of the generated EM field, allowing a 360° range of motion. A recent patent by Bucknor et al. [42] describes the development of an HMD fitted with EM emitters to track the movements of the user’s head when in a 3D virtual scenario. Such technologies can be exploited in the surgical scene to detect 3D virtual objects projected onto a patient’s body during robotic surgery, as in Pagador et al. [43], Liu et al. [44], and Diaz et al. [45], such that the handheld surgical tool emits EM fields when in communication with the HMD, for the augmented visualization of organs. In addition, the AR haptic system in [46] is calibrated to obtain precise tool coordinates within the global positioning system (GPS). Satellite technology improvements such as real-time kinematic (RTK) and high battery performance are required to increase the accuracy level per centimeter, as well as ML algorithms such as the Second Thales Theorem. As in most common tracking methods, EMTs are affected by visibility issues, occlusion, and the complexity of the algorithms used to register the workspace coordinates with the robot coordinate system. This form of tracking involves the inverse proportionality in the sensor–generator distance and its ferro-magnetic sensitivity, which tends to lower the output accuracy. This can be resolved by using EM tracking systems, such as in [47], where the RoboTracker performs the automated positioning and orientation of the patient without depending entirely on X-rays and conventional optics for accuracy.

(ii) Optical tracking systems (OTSs)

Optical tracking systems (OTSs) are extensively adopted in surgical navigation, the first proof of concept used during the Second World War, when optical sighting systems and gun detectors were a requirement for strategy planning. Zhou et al. [48] developed an infrared-based system with fiducial markers, integrated with a Bumblebee2 stereo camera lens for reduced optical interference during augmented viewing. According to Sorriento et al. [49], an OTS comprises a signal generator, a signal detector, and a control system, which processes the signal for accurate pose estimation. The operating principle of the optical tracking device includes determining the position of a 3D virtual object in space by connecting at least three visible and scattered points to form a known geometric pattern. Three non-collinear fiducial markers are required for the tracking of multiple end-effectors in six DoFs, for facilitated pose estimation. Double markers are used to detect positioning angles and the direction of the surgical tool tip when the values are independent of the orientation. During the clinical procedure, fiducial cues are rigidly registered to the surgical instruments and areas of interest to obtain location data in the range of 40–60 Hz, which is the most common frequency of human kinesthesia [50]. The information collated is then reconstructed by triangulation or back projection methods using mathematical algorithms such as geometric configuration, LED activation order, and displacement between sensors [50]. In video metric devices, pose estimation is determined by processing sequences of images from calibrated video cameras, albeit encountering background interference due to mechanical or optical stimuli [51]. However, IR-based optical tracking systems can perform multifunctional tracking using up to six active wireless tools, hence requiring lengthy computations and registration periods. This results in an increased cost for OTSs, which also plays a pivotal role in the overall system’s cost of manufacture and its market value.

### 3.2. Object Detection and AR Alignment for Robotic Surgery

Alongside the multitude of advances in other areas, such as dexterity and accurate image acquisition, commercial surgical robots are currently equipped with AR technology for the manipulation of resection tools. Their ability to visualize the patient-specific anatomy during affected tissue extraction allows them to work within safe workspace boundaries. While the precise mapping of medical images is unlikely due to the constant deformation of tissue pre- and post-surgery, many research papers [52,53,54,55] are dedicated to exploring the possibility of decoupling virtual objects and their sensory stimuli from the real world using algorithmic approaches adapted from the DL repository. Amongst the most acclaimed methods, projection-based AR, marker-based AR, markerless AR, and superimposition AR are widely used in robotic platforms employed in the operation theater and remotely. The section below provides examples of the types of AR tracking and the ways that they facilitate robot-assisted surgery.

#### 3.2.1. Intraoperative Planning for Surgical Robots

(i) Marker-based AR

**Figure 5:**
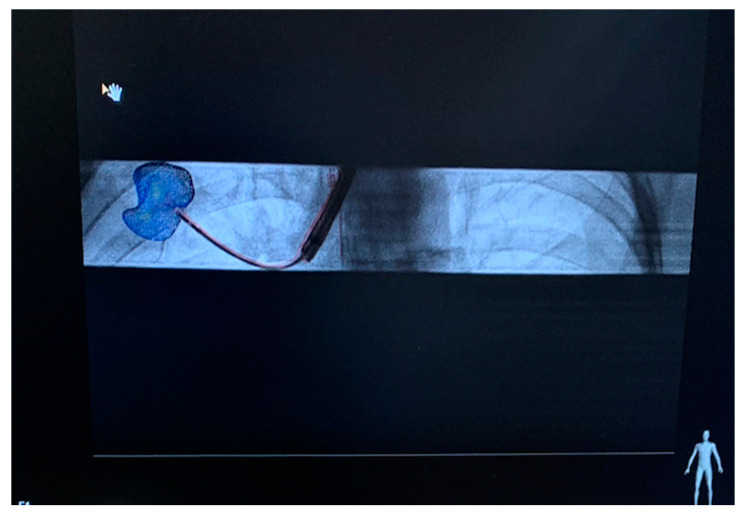
Intraoperative CBCT scan showing the pre-planned tumor location and position.

In marker-based AR technology, the main objective remains to drive a robotic system while performing coordinate estimation from the cameras relative to the markers. A plethora of marker sizes and types, with Vuforia and ArUco being the most popular, are utilized in a back-end working environment, enabling fluctuating marker information from the robot to be registered by the AR interface. This type of AR is useful in surgery that requires the triangulation of end-effectors to calculate their positions based on an added or moving fiducial marker with respect to the reference point (see Figure 5 for an example of a tumor biopsy using preoperative marker tracking using CBCT).

In the existing literature, Yavas et al. [56] used AR-based neuronavigation using optical tracking cameras such as LIDAR and AR light detection. Using markers within the operating scene, 3D preoperative registration and superposition is performed successfully with targeting errors between 0.5 and 3.5 mm, with facilitated placement of the fiducial marker. This application has been expanded by authors such as Van Duren et al. [57] and Luciano et al. [58], who created simulations of wire guidance through hip and thoracic screw placements using fluoroscopic imaging simulators embedded with orthogonal cameras to track virtual fiducial markers. Another widespread use of marker-based AR in preoperative planning and training includes haptic-feedback-enforced robotic simulators for midwives and novice obstetricians to perceive the required force during birth to exert during tool triangulation [59]. In cardiac support robotic systems, novices and surgeons alike can perform preoperative cardiac pulmonary resuscitation (CPR) through a series of training exercises on an augmented robot simulator, thus walking them through the core steps to perform when a patient’s heart stops [60].

(ii) Markerless AR

In its evolution from the detection of tangible markers in a surrounding environment, markerless AR registration requires no trigger for the detection of objects of interest in a workspace. Users can extract specific areas during visualization, estimate the corresponding transformation from the reference to image coordinate frame, and overlay the generated image above a real landmark. For instance, Liu et al. [61] recounted the utility of an AR-based guidance system for tongue tumor removal during transoral robotic surgery, where the daVinci robot gripper used collision avoidance algorithms to identify areas of interest and adjust the area of extraction in its holographic view. In [62], several proof-of-concept devices have been presented with low-cost digital platforms for vein location. They consist of high-intensity IR LEDs for virtual vessel enhancement diffusion on an augmented HMD or a smartphone such as the Google Nexus. Finally, Khuzagliyez et al. [63] described an AR-based visualization platform for the location of veins through ultrasound, using holographic-assisted, marker-free needle guidance for increased precision of cannulation. In a commercial setting, devices such as AccuVein (https://www.accuvein.com/why-accuvein/ar/ (accessed on 12 February 2023)) [64] and the NextVein Vein Finder (https://nextvein.com (accessed on 12 February 2023)) [65] are merchandized as wearable high-definition glasses that provide the smart, real-time 3D visualization of veins and arteries in separate layered views. This property reduces the risks of internal bleeding, patient discomfort, and patient–doctor codependence by 45% due to the constant monitoring through similar IR techniques. These devices all use markerless AR using the principle of digital laser scanning, hence improving the prospects of successful vein targeting by 3.5 times [66]. Kastner et al. [67] applied a markerless calibration technique in a HoloLens-based robotic system, which operated using point cloud data acquired from a depth sensor. Despite the slow processing time of the modified neural network controlling the movement of the robot, precise localization and augmented visualization was successfully achieved, albeit lowering the user experience. Another paper by Von Atzigen et al. [68] recounts the possibility of navigating a bending rod through a patient’s spinal cord after pedicle screw placement through CNN-assisted object detection techniques and AR-based axial orientation.

(iii) HMD-Based AR for Surgery and Rehabilitation

In the NAVIO Surgical System (Smith & Nephew, London, UK), which is built onto the HoloLens HMD, the surgeon performs intraoperative customized bone preparation and confirmation of the correct cut guide sizes through the overlaying of augmented drawings produced by surgical resection tools [69]. Moreover, a paper by Thøgersen et al. [70] introduced the concept of relieving patients from phantom pain, mostly experienced in amputees, after the loss of a limb or after a spinal cord injury. The real-time rendering of two healthy limbs in an HMD enables the user to perform specific actions in a game-like scenario while angular measurements of rotations are sent to a robotic platform from inertial sensors. Studies showed that the two participants who used this low-cost AR rehabilitation system for pain reduction immediately experienced lighter pain pre- and post-session. Further studies by Rothgangel et al. [71] depict the repetitive use of AR for analgesic outcomes on affected areas through a procedure called mirror box therapy. In these exercises, amputees can observe a reflection of the affected limb as intact through an avatar of similar appearance. They wear an HMD during the procedure and can mimic movement in line with the functioning one when using wheelchairs and robotic exoskeletons. These experiments are a turning point in the way that physiotherapists perform rehabilitation, such that patients can perform movement actions with AR-based software whilst supported by an exoskeletal structure.

#### 3.2.2. Preoperative Planning for Surgical Robots

(i) Superimposition-based AR

**Figure 6:**
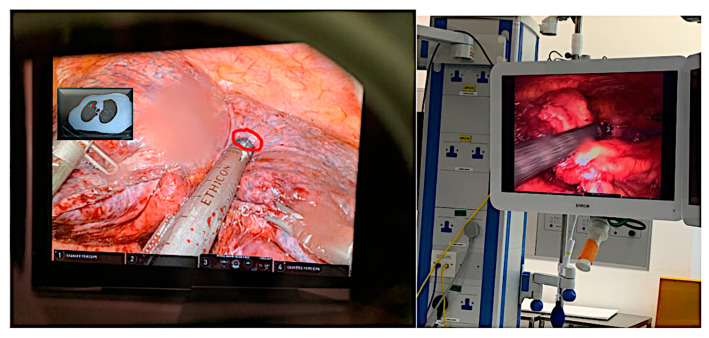
Superimposition-based AR tool navigation during right VATS segmentectomy and biopsy using HD monitor screens. The red marker indicates the correct positioning of the stapler to proceed with the dissection.

There are a wide range of applications for AR devices in the field of surgical robotics, which operate based on superimposition. The benefit of augmenting the surgeon’s FoV with reconstructed medical images and computer vision interfaces is primarily the ability to superpose useful information over real-world scenarios. This will enable an increase in efficiency in surgical setup and clinical arrangements. Widely appreciated by the surgical community, AR-based technologies include video-based displays that augment the surgeon’s FoV through video streaming [72], see-through displays that superimpose additional virtual objects onto the surface of the target user’s direct view [73], and projection-based displays that enable patient-specific models to be overlaid on patient skin, albeit reducing the geometrical accuracy and depth estimation. Liu et al. [74] described the use of a superimposition-based tracking system used to set up and register a digital reference frame of the craniofacial skeleton. The platform also consisted of an optical tracking system and a workstation to upload real-time data, which are transferred to the HMD for visualization. Pfefferle et al. [75] developed a renal biopsy system for needle tracking through tissue of interest by superposing holographic lesion representations from relevant CT scans (Figure 6). Moreover, Nicolau et al. [76] successfully introduced a miniature AR-based optical tube through a patient’s abdomen to visualize the endoscopic structures, which are not visible in direct camera view but are visible in the preoperative images. This type of 3D visualization is the first step in the development of a fully functional AR system, whereby the patient’s anatomy is transparent to the surgeon’s eye and important structures such as polyps, tumors, and blood channels can be identified in preoperative planning. This means that the control system operated by the surgeon can capture force feedback as well as perceive the approximate depth reached from the navigating channels, the results of which can be analyzed to create an interactive AR system for surgeons and novices alike. Salah et al. [77] described a similar approach for navigation along the spinal cord and the adjacent vertebrae, discs, and nerves using the in situ superimposition of reconstructed 3D models over the patient’s body.

Pessaux et al. [78] investigated the use of an AR-assisted robotic system to perform accurate incisions and detect areas of interest during robotic liver segmentectomy. Liu et al. [61] and Navab et al. [61] both praised the tool’s guidance during robotic surgery due to the ability to visualize real-time deformations with geometrical aids such as fiducial lines in their stereoscopic view. The authors in [79] used AR to reconstruct the external auditory canal and the tympanic membrane of the middle ear cavity as a 3D representation from preoperative CT scans. This procedure requires delicate navigation to prevent bleeding in the middle ear or perforation to the ossicles, therefore requiring accurate localization and an overall augmented view. Surgeons are then able to detect and target specific tissue, which may be difficult to ascertain or shift constantly. A novel approach to performing cancer biopsies, developed in partnership with KUKA Robotics and SIEMENS, has surfaced through the MURAB project [80], setting up a new workflow for magnetic resonance imaging (MRI) and ultrasound (US). Users can register the deformation of the target areas using relative force feedback and volumetric data. Such procedures ensure the precise targeting and extraction of miniature lesions with precise control under the guidance of an AR-based navigation system (https://www.murabproject.eu/about-murab/ (accessed on 22 March 2023)).

AR has also been used for the location of subcutaneous veins in preoperative surgery, as depicted in [70], where the proposed prototype has a USB camera connected to an Android smartphone to capture live frames of the vein using infrared sensors. The inherent ability of hemoglobin in the blood to absorb large volumes of infrared (IR) waves triggers the given phenomenon. The output images are then enhanced with contour filling [81], segmented through a thresholding technique, and are then displayed on a screen for superposition over a real-world anatomical landmark. Furthermore, Chen et al. [82] devised a mechanism to track the location of cues on the human pelvis by superposing a hologram of the latter over itself, using an HMD called the nVisor ST60 (NVIS Inc., Reston, VA, USA).

Another paper by Ma et al. [83] presented the use of AR systems for further preoperative planning before pedicle screw placement in spine surgery, a procedure that involved the use of ultrasound to generate 3D images from CT scans and then superpose them onto the areas of interest. Hajek et al. [84] used HoloLens to HoloLens communication to locate a bone in a user’s body; the devices were mounted on a C-arm fluoroscope and the patient, respectively. In studies by Elmi-Terander et al. [85], similar preplanning techniques were used to direct the drill trajectories for transpedicular screw placement using a cross-modality-based system called AR surgical navigation (ARSN). After procuring 3D CT scans of the surrounding spinal structures, the output DICOM data are warped into a 3D reconstruction of the spine, which allows for feedback-enhanced tracking to locate the areas of screw insertion. This is achieved by equipping the system with quadruple cameras, which are able to record a wider field of view from different angles and, in turn, display the 3D superposed images over the estimated drilling trajectory on a monitor. Moreover, the system proved to be reliable even during minimally invasive surgeries with percutaneous placement of Jamshidi needle tips at areas of interest to calculate the screw entry point and appropriate angles of insertion.

(ii) Projection-based AR

In projection-based (or spatial) AR, the areas of interest in the human body are displayed in a virtual world, without the use of HMDs and high-definition display screens. Using projection mapping, the augmented model of a landmark may be overlaid and dragged out of a screen using a tracking pen for realistic cardiac anatomy examination, as described in [86]. The available CT datasets were used to reconstruct the cardiac vessels and the associated separation between, for instance, pulmonary vessels of the hilum and vena cava, and visualize the behavior of a typical heart during grafting in a transplant. This method can be incorporated with a cross-examination using the computational model of the Total Artificial Heart (SynCardia Systems, Tucson, AZ, USA) in its virtual form, for accurate decision making, especially in terms of the biocompatibility of the scaffold heart in patients of younger age. Wu et al. [87] described the use of an AR navigation system to investigate the live deformation of surrounding tissue, nerves, and vessels via projections of the spine onto the patient’s back, which was reinforced with reference markers to overlay the image precisely over the patient. In other works, described in [88], the authors introduce the use of projector-based AR platforms to control a custom needle-guided robot using hand gestures over a preoperative ablation model projected over the area of interest. The Leonardo project by Simoes et al. [89] presented a similar interaction framework to plan the positioning of surgical ports by projecting the triangulation points over the patient’s torso.

(iii) HMD-based AR

According to Burström et al. [90], augmented reality surgical navigation (ARSN) techniques have been applied in the automatic position tracking of a given instrument to establish a real-time feedback loop of its location, leading to the enhanced identification of the virtual bone screw target point and angulation. After conducting several experiments, 97.4% accuracy was achieved during the extrapolation of the output data coordinates. Additionally, another robotic platform designed for spine surgery is MicroOptical (MicroOptical Corp., Westwood, MA, USA), which consists of an HMD for augmented intraoperative fluoroscopy performed in the vicinity of the internal fractures and the spinal pedicle screw placement. Out of the fifty case studies carried out on different patients, the operation time was significantly reduced due to the reduced view diversion from the patient. This also diminishes the rate of radiation absorbed by the medical professionals in the operating theater from the fluoroscopy generator. Furthermore, Lee et al. [91] describe an alternative to projection-based AR using a monitor screen to allow video sequence visualization in thyroidectomy to decouple the tactile feedback stimulus from the robot feedback system, during the resection of different anatomical structures. In a study by Agten et al. [92], the HoloLens was used to perform augmented visualizations of needle placements and insertions through a sawbone spine phantom made from opaque agar, as a simulation of lumbar joint punctures. After the reconstruction of the output CT scans of the phantom, the data were collated, loaded onto a headset, and projected onto the surgeon’s FoV as a hologram for precise needle guidance during the procedures, of which 97.5% were successful. Pratt et al. [69] conducted experiments to display and see through a patient’s inner vasculature in 3D during reconstructive surgery, through a HoloLens. The device is equipped with hand gesture recognition, enabling any hand movements to be captured, registered, and eventually converted into a hologram overlaying the existing lower extremities of the human body.

## 4. Software Integration

From the master–slave testbed to the operating theater, AR plays a pivotal role in the visualization of anatomical landmarks, particularly the ear, nose, and throat, as well as gastro-intestinal areas. AR-assisted robotic surgery has facilitated the surgeon’s task in reducing hand tremors and loss of triangulation during complicated procedures. Studies show that the transition from invasive open surgery to indirect, perception-driven surgery has resulted in a lower number of cases of tissue perforation, blood loss, and post-operative trauma [93]. In contrast to open surgery, which involves the direct manipulation of tissue, image-guided operations enable the surgeon to map medical image information, virtual or otherwise, spatially onto real-world body structures. Usually, the virtual fixture is defined as the superposition of augmented sensory data upon the user’s FoV from a software-generated platform that uses fiducial markers to register the location of an anatomical section in real time and space with respect to the user’s real-time scene. The use of publicly available datasets obtained from cutting-edge technology, such as CT and magnetic resonance imaging (MRI), in such scenarios enables minimal human error in data processing and hence improved success rates of surgeries.

### 4.1. Patient-To-Image Registration

The preliminary steps in diagnosing the area of concern in a patient include the use of computer guiding software to visualize inner anatomical landmarks. The loss of direct feel of the inner anatomy, reduced depth perception due to the monocularity of cameras, and distorted images have been addressed in novel techniques such as the segmentation of tissue in medical scans and 3D modeling for an augmented 360-degree field of view (FoV) [94]. In several papers by Londono et al. [95] and Pfefferle et al. [75], case studies of kidney biopsies examine the development of AR systems for the superposition of holograms over experimental phantoms. Studies show that preoperative CT scans from the lateral decubitus position result in deformed tissue internally, in addition to discrepancies between preoperative and intraoperative scans. Accurate image-guided surgery greatly depends on the registration of preoperative medical scans with their corresponding ones within the intraoperative anatomy. During the procedure, aligned coordinate frames are mapped onto the output registered image. The need to compensate for the time lag during registration means that multiple time frames are required at different regions of interest to enhance the quality of the registered image.

**Figure 7:**
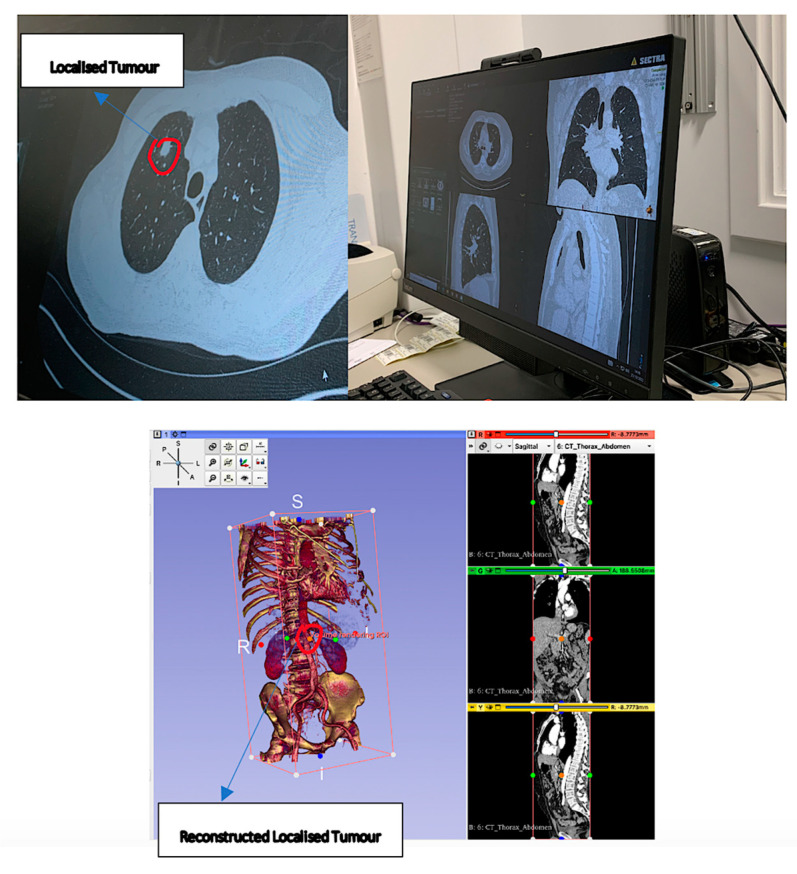
CT scans of the lung with its corresponding 3D reconstruction and marker localization, used by surgeons to locate tumors as indicated by the red marker.

Usually, the preferred choice of registration method depends on the type of robotic environment that the surgeon is navigating, where feature-based registration attracts the most attention within the academic community. These methods are less computationally heavy and can be used to effectively match fiducials between preoperative and intraoperative images, with primarily deformable methods of surface registration. Due to the sole use of 2D parameters, the possibility of obtaining highly accurate 3D information is low, hence driving the research community to establish novel sensing technologies for 3D marker tracking. Registration methods such as point-based registration, feature-based registration, segmentation-based registration, and fluoroscopy-based registration are widely used in the image processing of medical scans. The geometric transformations of deformable objects are computed using fiducial markers, which act as positioning cues and can be analyzed for fiducial localization errors (FLEs). In cases where images have varying gray levels, DL algorithms are able to segregate different features using parameters such as the sum of squared or absolute differences (SSD), correlation coefficients, and mean squared difference (MSD). For real-time X-ray image processing, a contrasting material, such as barium or iodine, is used to create more subtle contrast differences for clinicians to analyze. The process of 2D to 3D image registration involves the alignment of matching preoperative and intraoperative features, which can be reconstructed in AR and superposed over a live fluoroscopic image with respect to reference points in the image sequence (Figure 7).

### 4.2. Camera Calibration for Optimal Alignment

Automatic camera calibration and corresponding image alignment in intraoperative ultrasound is used to determine internal structural characteristics such as the focal length and surface anatomy of different organs. Analysis, visualization, and pre-planning using registered medical images enable the development of patient-specific models of the relevant anatomy. The researchers in [96] created a cross-modality AR model to correct the shifts in positioning using lesion holograms, generated during a CT image reconstruction process. A US transducer obtains two-dimensional scans from the site of interest and is merged with magnetic tracking data to produce a 3D resultant scan in line with a CNN algorithm. This alleviates the probability of false negatives appearing in the dataset, especially when mapping magnetically tracked ultrasound scans onto non-rigidly registered 3D scans for the detection of mismatches in deformation. Furthermore, this method is also used for needle guidance, as mentioned in [75], to predict trans-operative pathways during navigation, as well as detecting areas of extraction for lesions on Unity3D via the collision avoidance system. The object-to-image registration is optimized by placing markers, sufficiently far apart in a non-linear configuration, such that their combined center coincides with the projection of the target in the workspace.

### 4.3. 3D Visualization using Direct Volume Rendering

The next steps in creating an AR model include image processing techniques such as direct volume rendering, which are used to remove outliers and delineators from raw DICOM data. A method proposed by Calhoun et al. [97] involves voxel contrast adjustment and multimodal volume registration of the voxels in the CT images by replacing their existing density with a specific color and enhancing their contrast through thresholding, performed by a transfer function. Manual intensity thresholding removes all low-intensity artefacts and background noise from the image, ready for rigid attachment to an organ in virtuality. A transparency function is applied to filter out extreme contrasts in anatomical or pathological 3D landmarks and any blob-like contours detected can be used in the initial registration of CT scans under techniques such as topological structural analysis. The deformation properties of the organs are modeled using software such as Osirix 12.0, 3D Slicer 5.2.2, or VR-Render IRCAD2010, and the high contrast applied to output images makes structures such as tumors, bones, and aneurism-prone vessels more visible to the naked eye.

### 4.4. Surface Rendering after Segmentation of Pre-Processed Data

Surface rendering techniques in [98] depict the conversion of anatomical structures into a mesh for delineation and segmentation. Tamadazte et al. [99] used the epipolar geometry principle to acquire images from the left and right stereovision cameras. The authors then used a point correspondence approach to resample and build a 3D triangular mesh from local data points in its neighborhood. The current techniques utilized in AR are developed using a software program called Unity3D and require patient-specific polygons such as triangles for rapid processing. Furthermore, the anatomical scenes detected using US transducers may be reconstructed using multi-view stereo (MVS), which analyzes pieces of tissue extracted from an area, remeshes them by warping the raw DICOM data, and displays them with appropriate textures using speeded up robust feature (SURF) methods [100]. In most cases, segmentation may cause the loss of essential information in the original volume data. Therefore, in the quest to improve the quality of segmented images, Pandey et al. [101] introduced a faster and more robust system for US to CT registration using shadow peak (SP) bone registration. In another study by Hacihaliloglu et al. [102], similar bone surface segmentation techniques have been used to determine the phase symmetry of bone fractures.

### 4.5. Path Computational Framework for Navigation and Planning

In studies by El-Hariri et al. [103] and Hussain et al. [79], the use of tracking mechanisms for marker-based biopsy guidance has been widely commended and applied in surgery, such as that of the middle ear and the kidneys. Fiducial cues are registered to different locations on the patient’s body, using the robust surface matching of sphere markers with the standard model, alongside laparoscopic video streams. Image-to-patient registration is performed by comparing the acquired live images to the available patient-to-image datasets, which is a crucial operation to eliminate errors during automatic correction, as explained by Wittman et al. [104]. Leeming et al. [105] used proximity queries to detect internal changes in anatomy during the manipulation of a continuum robot for surgery around a bone cavity. A covariance tree is used in this case, as a live modeling algorithm, to maintain an explicit safety margin between the walls of an anatomical landmark during the maneuvering of surgical tools. For cases of minimally invasive surgery, precautionary measures such as CO_2_ inflation of the patient’s body and highlighting target locations with contrasting colors (for example, with ICG) facilitate the surgeon’s task, especially when performing cross-modality interventions with AR systems such as headsets. A study by Zhang et al. [106] explained the tracking mechanisms used in US procedures for intraoperative use. The probe was equipped with a HoloLens-tracked reference frame, which contained multiple reflective spheres on an organ. In terms of biopsy needle tracking, Pratt et al. [81] introduced the concept of registered stylus guidance in line with a simulated 3D replica reconstructed from CT images of the torso. During preoperative surgical navigation, a calibrated probe is used to collect data from internal organs to send to the 3D Slicer software over OpenIGTLink, whilst combining tracked data from the input instruments. The stylus tip is calibrated about a pivot and can be moved to various positions in the anatomical plane while tracking it over the probe reference frame using an iterative closest point (ICP)-based detection algorithm. Jiang et al. [106] proved that the projector view for puncture surgery also improves the efficiency of perception-based navigation, using superimposed markers to align the needle tip to a magenta circle. The researchers in the above study generated an accurate AR positioning method using DL techniques such as the Newton–Gauss method and Lie algebra to produce an optimized projection matrix. Any projection is performed towards the target location of the body, hence reducing the probability of parallax errors, as shown by Wu et al. [107].

## 5. Applications of Computer Vision in Surgical Robot Operation (DL-Based)

With the groundbreaking development of artificial intelligence (AI) in assistive surgical robots, the healthcare sector today has seen a booming increase in the data collected and stored in databases, such as in the NHS. During the COVID-19 pandemic, technology has lessened the burden of healthcare workers behind the scenes, minimizing the need to sort, collect, and store data manually, as well as cutting down costs in decision-making tasks. Training datasets for early symptom recognition, estimating patient mortality rates, and abnormality detection in specific tissue images have enabled researchers to obviate error-prone concepts during robot training and prepare novices for unexpected fallacies [108]. The surgical community has recognized the pivotal role that AR integration in DL-based robotics plays, including increasing the transparency of the patient, higher accuracy, less bleeding, and shorter recovery times. The possibility of reducing the exposure to harmful radiation and pathogens has also proved beneficial for overall surgical efficiency in clinics, especially in the post-pandemic world. Despite the multitude of benefits that AR presents, there are still a number of issues that have been identified, as in Table 1, such as incorrect interposition and mapping between real and virtual worlds, the inaccurate visualization of organs of interest due to difficulties in estimating their positions, and a lack of correspondence between the real tissue and the virtual tissue. The projections of AR-based reconstructions may be inaccurate at times due to various real-time factors, such as the indefinite structures of internal organs and boundaries, fluctuations in vital signs, and subtle human body movements such as aspiration and blood pressure [109].

The introduction of the DL-based optimization of surgical robot performance, according to Govers et al. [110], enables intelligent task planning and operation, in contrast to manual robotics, which only applies pre-defined output reflexes. These intelligent robots are environment-aware and can perform perception-based obstacle-aided navigation, for the shortest displacement decision making within restricted passageways. According to Conti et al. [111], physical robots that have embedded lasers, IR cameras, and ultra-wideband radios can be trained using DL algorithms to track human–robot interactions in augmented environments. Zhang et al. [112] described the use of AR to control physical interactions, achieve sensor-based navigation, and perform complex trajectory planning using DL methods under changing external stimuli. The following section provides an overview of the DL algorithms used to increase the efficiency of robot performance and end-effector positioning accuracy in proof-of-concept robotic platforms.

### 5.1. Medical Image Registration

Recently, papers by Garon et al. [113] and Alhaija et al. [114] have described the implementation of DL algorithms such as CNNs to allow marker-based image registration within given parameters (See Appendix B). An AR-modified neural network is proposed for efficient object detection and point cloud extraction in line with the ComplexYOLO architecture. Another paper by Qi et al. [115] proposes a different neural network, known as the PointNet network, for semantic segmentation as well as 3D object localization within raw point cloud data. Estrada et al. [33] depict an array of deep neural network architectures to train large datasets without the need for feature engineering. DL methods such as region-based CNNs (RCNN) [116], you only look once (YOLO) [117], and single-shot detectors (SSD) [118] have been applied in several works pertaining to surgical image registration. Extending from this concept, the popular SLAM algorithm, classified as feature-based operations and direct operations, can be used in the localization of anatomical defects. Feature-based methods focus on the principal image locators or features, whilst the direct method utilizes the data from each pixel in the image to determine the parameters of the target image posture. The studies by Klein et al. [119] and Mur-Artal et al. [120] describe the use of monocular feature-based tracking using a real-time pose estimation system called Parallel Tracking and Mapping (PTAM), as well as an alternative called ORB-SLAM. These algorithms reduce the batch operation period and create a large coordinate system within keyframe constraints for more accurate pose estimation.

### 5.2. Increased Optimization of Robot Orientation Using Motion Planning and Camera Projection

The position and orientation of surgical robots are determined by the linkage arrangements and their relative degrees of freedom. In each workspace, the configuration of the manipulator is specified for each joint to allow inference of the position of any variable. According to Adhami et al. [121], the concept of AR can be applied to determine the DoF of a manipulator according to its configuration space using a systematic method of positioning surgical robots with high n values to optimize their performance. Recent experiments conducted by Gonzalez-Barbosa et al. [122] and Yildiz et al. [123] depicted the use of optimal camera placement for the wider angular coverage of a specific workspace using a camera projection model, two-step strategies for robotically assisted minimally invasive surgery (RAMIS), and deep learning algorithms such as Wireless Video Sensor Networks. Similarly, Gadre et al. [124] utilized the Microsoft HoloLens as an interface for the visualization of a target curve for a real Baxter robot. Furthermore, studies by Fotouhi et al. [125] made use of Kinect sensors in their experimental setup to register images of their robot from multiple angles, which were used to determine the accuracy in AR alignment. The use of a DT of the KUKA robot in motion enabled the surgeon to estimate the correct position and orientation during an operation, via a reflective AR mirror. The accuracy parameters depended highly on the precision taken in reconstructing medical images, with a 33.7% success rate.

### 5.3. Collision Detection during Surgical End-Effector Motion

Once the topology of the robot is achieved, the orientation profile is checked for the collision-free volume (CFV) using a swept volume visualization process, as described in [126]. A sequence of control coordinates is selected on the contour of the output profile to specify the pose of the robot arm at each control point. The use of an AR interface enables efficient CFV mapping and collision detection among the registered virtual models. In recent papers, such as [127], self-collision detection checks are performed using V-COLLIDE, where the robot links are converted to STL format. The end-effector is only considered collision-free when the swept control points are within the CFV range, which is visualized by the user via projection-based AR devices. Determination of the CFV range is particularly essential for precise port placement in robotic-assisted laparoscopic surgery [128], where collision avoidance allows for maximum port access and the visualization of areas of interest. To reduce the number of cuboids in each reconstructed mesh before a collision detection procedure starts, several algorithms, such as the tight-fitting oriented bounding boxes (OBB) and axis-aligned bounding boxes (AABB) algorithms, are applied. They are used to calculate the shortest colliding distance in convex polyhedral collision models [129]. Zhang et al. [130] described the qualitative results of tissue reconstruction from the surface meshes of point clouds to the anatomical margin of interest. The experiment proved that the fast collision method used on the OBBs after automatic cube tessellation achieved a feedback rate of approximately 1 kHz, hence able to provide unparalleled control during robotic surgery. Coste-Maniere et al. [131] describe the possibility of AR-based collision detection and the increased accuracy of virtual tool placement within flesh, ribs, and target locations. The use of a heatmap superposed over a patient’s body has also been explored in works by Weede et al. [132], to calculate the goodness value of the plane. The authors in [133] discuss the evaluation and calibration of such robotic systems, which showed a relatively high degree of accuracy, albeit with a few hindrances in terms of virtual EE alignment with the lectern interaction tools due to limited DoF. These methods are, however, highly successful in contour tracing and profiling to produce a virtual smooth and collision-free workspace along the output curve. A list of existing techniques in collision detection is tabulated below for readers to compare the most efficient learning methods used to attain accurate trajectory planning (Table 2).

### 5.4. Reconfiguration and Workspace Visualization of Surgical Robots

Most malleable robots in surgical settings require accurate port placement and end-effector positioning, adapting to the desired user requirements within a specific workspace reconfiguration. Each revolute joint of the robot can be aligned and positioned using augmented visual cues, hence guiding the user towards the required robot topology. This method is gaining popularity in surgery due to the ease of motion tracking and calibration, with demonstrated accuracy of up to ±2 mm. In works by Ranne et al. [140], an AR-assisted robotic system with OptiTrack sensors is implemented for the smooth generation of a virtual end-effector, which is placed in its maximum reachable space. The computation of workspace configurations can be performed individually, which generates a virtual cue in the user’s FoV. Previously, a VR platform developed by Lipton et al. [141], called Baxtor’s Homonculus, introduced an intermediate virtual scenario for the mapping of the robot reference frame to the user’s, decoupling the sensory stimulus from the translations of end-effectors. However, it has been observed that the mapping of the robot’s reference frame onto that of the user may be problematic, due to inaccuracies in the alignment of the end-effector in virtual-to-real scenarios. With the aim of increasing the precision of such mapping, Bolano et al. [142] used point cloud extraction to predict robot–end-effector collision during the swept volume visualization and orientation profiling of the robot. The algorithms used for the mapping of the P3-5 end-effectors of a robot arm are explained in detail in [143], emphasizing the accuracy of inference and virtual feedback from the HoloLens with respect to the origin. The user is given real-time feedback on the alignment error between the current and desired position based on the mesh model generation of a link and translation around the reference end-effector. Other applications of AR in the orientation planning of end-effectors are described by Gao et al. [144], who investigate the optimum inclination angles of a robot linkage whilst following a visual cue with respect to a particular path. Human–robot interaction is smoother at optimum angles as display–control misalignment can be reduced and precise port placement can be selected during an operation without risking patient safety.

### 5.5. Increased Haptic Feedback for Virtual Scene Guidance

The applications of the daVinci research kit have been reported to be broad in the academic community, ranging from collaborative research in RAMIS to independent surgeon manipulation using a stereoscopic system [145]. The development of an ROS interface has been the stepping stone in initiating a novel motion planning framework in line with haptic feedback. This relies mainly on MoveIt and the Fast Collision Library (FCL), which are currently used to upload a specific mesh object in simulation and check for collisions in the panned PSM environment. According to Zhang et al. [145], the simulated PSMs produce deflections, which are fed through as input and produce sensing feedback at the main manipulator system. The direction of velocity for each PSM end-effector, normal to the surface, v, is instead utilized and described mathematically in the equations below: when the surfaces U and V approach a point coordinate such that ∆ (U,V) converges to a zero value, the surface normal becomes collinear and this feature may be expanded in order to create a spherical proxy region (SPR) at the end-effector, which is the target area for interactions.

In the existing literature, a multitude of haptic sensors have been proposed to enable high-speed performance in data pre-processing and rendering. The stereotaxy phenomena in surgical scenarios such as gamma-knife surgeries [146] create an illusion of registered and reproduced 3D haptic feedback data in the form of a sensory stimulus. In a paper by Srinivasan et al. [147], the effect of visually locating markers was investigated, which allowed a correlation to be made between the perception feedback of the cues and the actual haptic feedback obtained from an object. The textures of specific objects rendered in a virtual world were studied by Basdogan et al. [148], to allow for the creation of tangential frictional force-generated textured surfaces in line with a technique called bump mapping. Furthermore, a review published by Latimer et al. [149] described the behavior of several polygonal rigid bodies during haptic interaction, as well as the challenges of their forward collisions on surrounding forces. More recently, research by Costa et al. [150] generated a simulated environment for anatomical tissue using the long elements method to estimate object deformation in a gravitational field. Such theories can be widely exploited in the world of AR-assisted surgery to better understand the behavior of end-effectors within the bodily vessels both in real time and virtually.

In a surgical setting, works by Okamura et al. [151] and Westebring-Van der Putten et al. [152] have been commended for their research into various haptic devices, the types of interaction control, as well as the intelligent proxy-based algorithms used to assess deflections or collisions in a proxy workspace. Additionally, Wurdemann et al. [153] presented a novel wearable device that could provide accurate haptic feedback, and Li et al. [154] adapted this design to apply the pseudo-haptic feedback (PHF) technique for hardware-free experimentation using visual cues. A field of surgery that requires considerable haptic implementation is plastic surgery, which requires the overlaying of virtual images for surgical guidance, such as in Tsai et al. [155], who used a haptic device to deflate a protruding zygoma and for implant positioning during facial contouring. In works by Schendel et al. [156], 3D visualization was used for the surgical planning and manipulation of patient skin models for a cleft lip repair surgery, in accordance with the output from haptic devices. The application of AR for cranio-maxillofacial complex fracture reduction has been explored by Olsson et al. [157], whereby the patient’s bone mesh models are generated and an immersive experience is created using software such as Unity3D 2023.1.2 for accurate end-effector guidance in educational training.

### 5.6. Improved Communication and Patient Safety

Robotic surgery has improved the way in which surgeons gain access to the difficult internal anatomy, bringing significant advancements in transmission latency from the first transatlantic robotic-assisted laparoscopic cholecystectomy performed in the late 20th century [158]. With the advent of 5G mobile communication technology, the field of AR in surgery has been revolutionized, with an increased ability to perform MIS via fiber optic technology, for example, at a cheaper cost and more widespread throughout clinics worldwide. The use of smartphone applications for teleconferencing has been widely recommended by surgeons operating on robotic platforms, for instant access to web-based resources as well as near-ubiquitous peer–doctor communication. This is especially useful in the post-pandemic world, where the immediate advice of off-site staff is required, such as in cases where a visual review would be beneficial for injury assessment. The development of smart wearable devices such as HMD-based systems allows the user to obtain reliable audio-visual data with minimal latency during an intervention due to the fast transfer of data through 5G networks [159]. These devices also help surgeons to perform simulations and adopt extended reality scenarios when mentoring novices and remote colleagues during various surgical procedures, by using models of surgical specimens and case studies. Recent studies have shown that any latency experienced during robotic surgery, remote or otherwise, increases the risks of bleeding and mistargeted tool placement, which may lead to complications. Moreover, 5G technology enables the relaying of haptic feedback to the surgeon in real time, through gyroscopic all-motion cognition as well as tactile sensors. This provides a sense of real touch for the determination of the depth, precision, texture, and contours of tissue and organs. AR technology can facilitate the learning and simulation of tissue resistance techniques, such as determining the weight and force required to insert and remove a needle. There are also possibilities to incorporate DL algorithms within the learning database to adapt the system for performance analysis with supervised memory during simulation-based training and assessment.

The link between communication and patient safety has been highlighted in works such as [8,12], where the different types of surgical hazards, such as trajectory misjudgment and diversion, electrical faults, and time lags, can pose serious risks for the patient. Hence, AR displays play a pivotal role in overcoming the effect of the lag between the remote operator and the robot platform, providing instant visual feedback to the user. As established by a clinical trial [160] performed at IRCCS San Raffaele Hospital in Milan, the efficiency of remote proctoring in guiding the implantation of medical devices is significantly increased. A combination of AR visors, 5G telecommunication, and multi-access edge computing (MEC) enables the surgeon to access live medical imaging and a holographic model of the human heart directly from the operating theater, through a low-latency 5G network. The paper by Richter et al. [161] explains the use of a stereoscopic AR predictive display (SARPD) to display the preoperative displacements within anatomical margins, eliminating the risk of overshoot and oscillations in navigation. The use of an extended Kalman filtering (EKF) framework enabled visual alignment between predicted and actual movements using kinematic calibrations. Ye et al. [162] described an experimental setup that facilitated error-free hand–eye coordination during end-effector placement, whilst successfully rendering augmented objects such as slave–tool systems and geometric fiducial cues. According to Takahashi et al. [163], the pre-transmission compression of surgical images requires an acceptable level of delay before the irreparable loss of anatomical data. Generally, a delay time of up to 140 milliseconds can establish sufficient connectivity for minimum data loss and image compression. Despite the advancements in runtime for robotic platforms such as the DaVinci, achieving up to 36 fps on AR displays, we emphasize the need for further study on the accuracy of measurements as well as the rendering pipeline to reduce the cognitive load during tool manipulation.

### 5.7. Digital Twins (DT) to Guide End-Effectors

In the post-pandemic world, medical DT provide the integrated and virtual visualization of patient data and hence create a user-friendly software platform for surgeons to access complex information such as physical, physiological, and cognitive characteristics. DT can play a pivotal role in remote patient monitoring through advanced diagnostic tools, whilst incorporating AR for precision medicine, for a more patient-centric method of treatment. The core purpose of DT in AR-based robotic surgery is personalized medicine using patient-specific modeling from deep learning databases to accurately determine the cause and treatment of a disease. The table in [164] summarizes the various applications of DT in the surgical field from 2011 to the present, with some notable systems cited, such as the Philips Heart Navigator Tool, which combines CT scans from different angles of an organ and generates a real-time 3D model for the accurate positioning of surgical tools and faster preoperative planning. The need for the data-driven control of dosage effects as well as device responses before treatment has risen, being important to predict the behavior of patients after heart disease management, such as in works by Niederer et al. [165], where mechanical models were used to investigate the effects of cardiac resynchronization therapy (CRT). The use of DT to treat cardiovascular diseases through semi-active modeling of the heart, with real-time blood flow and head vibration, facilitated the localization of stenosis in a modeled human face. In other surgical uses, the company Sim & Cure employed 3D rotational angiography to generate an interactive model of an aneurysm, to direct the tools towards the ideal implant coordinates [166]. The implementation of a cloud GPU, computer vision, and ML technology enables the augmented visualization of anatomical landmarks and blood circulation, through a DT model. The efficiency of surgical performance is increased through combined simulation and AR platforms, hence significantly improving the training graph via an increasing optimization gradient. Other DT surgical models include post-operative bone structure modeling in 3D from CT scans, which enables accurate rotation and imaging system orientation through the compensation of the subtalar joint axis, as explained in Hernigou et al. [167].

Owing to its high success rates in industrial robot integration, the authors in [168] have established a proof-of-concept AR-integrated system for surgical interventions based on ROS and Unity3D. The work was based on the lightweight KUKA robot, which can be manipulated using the TMFlow software in parallel with pre-defined programs. The robot was controlled using Python, where scripts such as *MqttOmronMovePublisher* package the joint angles and publish them on the /omron/command MQTT topic, which is then received by the /omron/move/command at the ROS side after conversion and JSON deserialization. This information is then received by *MqttOmronMoveSubscriber*, which attaches these joint angles to the corresponding robot arm and enables the DT controller to achieve the desired configuration. In this way, the OMRON robot can perform multiple movements whilst visualizing them on the ghost robot in the background. In another study, a similar virtual-to-real mapping technique was used to simulate an abdominal surgery, estimating the virtual-to-real 6-DoF robot’s alignment within an AR environment [124]. The integration of reflective AR mirrors enables a simultaneous view of the scene from various angles, whilst images are captured by a camera sensor on the HMD to be analyzed for alignment accuracy. The Euclidean distance between the reference frame of the camera center is mapped to that of each joint by colliding the cursor with the AR workspace. The errors achieved between these joints were compared using the fixed, reflective, and single-view displays, with a misalignment error of 16.5 +/− 11.0 mm, which was lower than when no reflective mirrors were used.

Nowadays, most DT technologies enable AR integration in order to support model adjustment based on user feedback, immersion, and intuitiveness. For example, the ARAILIS prototype in [169] provides calibrated digital software for AR mapping and image segmentation, via a SLAM algorithm for object detection. The ROS2 middleware enables communication through a modular architecture, allowing for safety and privacy encryption. Furthermore, the output from the calibration process increases the precision of the ORBSLAM algorithm for the supervised learning of the real-to-virtual world coordinate mapping. The human-in-the-loop collaboration with robotic DTs is a crucial requirement for dynamic modeling and data annotation—for example, to locate tumors, detect misalignments, and transpose 3D models. This enables a multi-user system to be set up for other medical bodies to refer to as a constant knowledge database, through a human–machine collaborative intelligent platform.

## 6. Discussion

Despite the prevalent breakthroughs in AR for robotic surgery, there are several pitfalls that have been reported by users of such technology, which need to be addressed. Several papers have been reviewed in the existing literature, addressing the core gaps in the field and potential improvements in the efficiency of robotic platforms. These papers were classified in terms of hardware, software, and DL applications, with a total of 170 papers. We realized that several papers lacked a focus on accuracy in feedback systems, alignment during interactions, registration techniques, and patient safety for robots of varying LoA. We also identified difficulties in sight diversion during hand–eye coordination for a surgeon, which means that rather than switching between real and virtual scenes, an integrated lectern or platform can be used for the experiments instead [169]. Employing virtual monitors through the HoloLens reduces the discomfort of constant view diversion and allows greater situational awareness, as in the case of Yang et al. [170], who utilized color-coded margins to optimize the resection margins. In the case of occlusion, it is suggested that the field of view for the operating surgeon and the spatial relationship of individual landmarks be optimized so that only one plane is visible at a time. This would significantly reduce the clinician’s cognitive load during a surgery, as well as improving the depth perception of internal organs and reducing visual clutter and the latency of the entire surgical system. Any visual clutter produced by excessive data in a surgeon’s FoV may risk the safe placement of the robot end-effectors around the sensitive anatomical walls. A transparent AR model with varying window levels enables the successful navigation of the tools from the skin layers to the bone.

The integration of AR in surgery requires the precise calibration of the end-effectors to localize the coordinates of the objects of interest within the workspace. Fiducial markers are predominantly used in object localization on medical scans, especially in the pre-planning of convoluted navigation procedures. Techniques such as the rigid and non-rigid registration of such markers, using image deformation methods to move tissue, enable the accurate reconstruction of unstructured robotic environments, allowing for the generation of a historical trajectory map. In the image registration stage, the accurate transferring of matching features from preoperative to intraoperative images requires virtual potential fields to reference the locations of areas of interest, such as tumors or blood vessels. The use of manual registration techniques before a fully autonomous navigation procedure allows the elimination, to an extent, of any errors that may be caused by misleading situational awareness, as in the two-way DaVinci robot registration. The integration of DL methods at this stage of validation and testing helps the system to learn the correct position of the defect within an exhaustive database, hence ensuring higher repeatability.

Despite the plethora of advantages that the development of CNN databases for AR navigation provides, our studies showed that the feasibility of many procedures is hindered by human errors, such as the movement of the patient, the rapid deformation of the tissue, such as the lung, and the instability of clinical equipment, as well as high levels of background noise in captured medical images. It is therefore of the utmost importance that researchers bridge the gap in alleviating these challenges by employing more efficient deep learning algorithms, such as image deformation methods, to parametrize the no-risk zones within an anatomical space. Registration algorithms such as the head and hat algorithm and the iterative closest point (ICP) algorithm facilitate the extraction and geometric transformation of specific 2D or 3D surfaces on deformable multimodal images, based on neural network architectures (CNNs, RNNs, and LTSMs).

The ability to detect collision-free zones for accurate end-effector placement requires complex calculations of workspace reconfigurations, whilst plotting the coordinates of the output curve within the safe margin of interest. The authors in [115,129] have largely inspired the extraction of point clouds to create mesh models of the volume swept, hence enabling the user to visualize the exact maximum reachable space within which the robot end-effector can reach. There is a need for the further development of such algorithms to enable a correlation between real-time deflated images from dynamic organs such as the heart and the lung, with respect to their marker counterparts on inflated medical images. In these cases, the integration of a digital twin (DT) can become useful, to reflect and visualize the robot motion whilst performing the operation. The ghost robot allows the user to view the final configuration of the robot in RoboDK in a pre-planning stage, which enables corrections to be made, if necessary, both in the simulated environment and via the MQTT LAN bridge, which connects Unity3D to the collaborative robot.

The legal and ethical aspects of AR in surgery have been debated in several courts, due to the skepticism that comes with performing operations from a distance or even cross-territory. The process of the clinical evaluation and validation of AR-based surgical robots remains in its early stages of development, and experiments performed on phantoms such as 3D-printed organs followed by cadavers and patients are claimed to have the highest rates of accuracy [171]. However, due to the lack of evidence of the increase in surgeon comfort or clinical performance in such validation techniques, further research focused on larger patient datasets, higher precision in 3D reconstruction, and depth perception may enhance the outcomes of AR-ready clinical evaluation. Despite the ongoing criticism, the current success of AR in surgical education may also encourage further research into faster and more accurate robot performance [8]. Although in its early stages due to a lack of objective metrics to assess its impact, this application can further improve the performance of various Level 1 and Level 2 robots (see LoA in Section 1) under the patient safety guidance and ethical approval [12]. We also noticed an increase in FDA clearance under the CE marking of devices utilizing AR in surgery, which is a promising aspect towards AR-assisted robot deployment in hospitals.

## 7. Conclusions

This paper provided a general overview of various surgical robotic platforms available on the market and in the existing literature, with an emphasis on their system architectures, software platforms, and learning methods. A total of 170 papers were selected and analyzed in a systematic manner for the purpose of identifying the relevant literature that described the types of AR technologies used in surgery. AR remains a promising tool in facilitating the surgeon’s task, from docking the station to port placement and end-effector guidance. To counteract the difficulties experienced by manual operation, AR visualization helps surgeons to perform interventions efficiently through HMDs, spatial projectors, and marker-based or markerless interfaces. This review focused mainly on the plethora of AR interfaces used in surgery, focusing on three main aspects: “hardware”, “software”, and “application”. The roadblocks towards achieving optimum AR integration were addressed and a wide range of solutions was presented to increase the efficiency of existing robots. The ability to eliminate visual clutter and occlusion within the surgeon’s FoV opens the door to novel augmented models with different layers and windows, which can be chosen according to the degree of importance. In areas such as thoracic surgery, gynecology, and plastic surgery, where the haptic feedback system provides an indication of the type of force required for an intervention, we found that the use of AR integration with force feedback sensors, as in the DaVinci master–slave console, increases the sensory stimulus of the surgeon, with a direct correlation between the fiducial cues and the real-time feedback from the object of interest.

Owing to its popularity amongst surgeons, AR is widely commended by the research community for surgeries ranging from tumor detection to vein location through fiducial markers, despite its restrictions in terms of spatial awareness and occlusion. To reduce the risks of bleeding in conditions where the surgeon is required to coordinate hand–eye movements, we introduce the novel concept of reflective AR and DT technologies, which are in their pilot stages. The level of accuracy in areas such as suturing, knot tying, and pick-and-place operations has significantly increased as compared to manual operations, which inspires further research in this sector. At the time of this literature review, to the best of our knowledge, there exists a limited pool of specialized papers in the field of AR for surgical robots containing a detailed rundown of novel AR platforms with DL algorithmic approaches. Our paper aims to identify the research gaps in areas such as hardware components, software specifications, and the application of DL in various surgical stages. We believe that we have laid the foundation for the future of AR for surgery, which will not only be useful for researchers but also surgeons and novices who wish to learn about AR-assisted surgical interventions with accurate tool placement, without limiting the reader to previous conventional trends in the sector.

## Figures and Tables

**Table 1:** Technical bottlenecks in the field of AR according to Roger’s theory.

Technical Bottlenecks	Description
Compatibility with social practices	Wearable devices such as Google Glass may create privacy issues.
Complexity (user-friendliness or learning)	AR is easy to learn by novice surgeons and can increase the learning curve.
Lack of accuracy in alignment	Modern DL algorithms such as deep transfer learning and supervised and unsupervised learning are used to tackle the issues in real-to-virtual world mapping. Lighting conditions can be adjusted for better alignment.
Trialability to general public	Easily deployed but may be expensive to test in several regions simultaneously.

**Table 2:** Types of collision detection techniques and corresponding learning methods used during path planning.

Author(s)	Collision Avoidance Technique	Learning Method	Accuracy
Wang et al. [134]	Zero robot programming for vision-based human–robot interactions, linking two Kinect sensors for retrieval of robot pose in 3D from a robot mesh model.	Wise-ShopFloor framework is used to determine initial and final pose.	N/A
Du et al. [135]	Fast path planning using virtual potential fields, representing obstacles and targets, as well as Kinect sensors.	Human tracking using unscented Kalman filter, for mean and variance determination of a set of sigma points.	Lower avoidance time (>689.41 Hz).
Hongzhong et al. [136]	Preliminary filtering of mesh models to reduce the number of cuboids in experiment. Virtual fixtures known as active constraints used in generating resistive force. Automatic cube tessellation used for 3D point detection and collision avoidance.	Use of oriented bounding boxes (OBBs) and filtering algorithms: Separating Axis Test and Sweep and Prune. Use of field-programmable gate arrays to design a faster GPU system.	Frame rates of 17.5 k OBBs using a bit width of 20, update rate of 25 Hz compared to 1 kHz.
Das et al. [137]	OPML motion planning using standard geometric collision checkers such as proxy collision detectors.	Learning-based Fastron algorithm used to generate robot motion in complex obstacle-prone surroundings.	100-times faster collision detection than C-space modeling.
Torres et al. [138]	Concentric tube robot teleoperation using automatic, collision avoidance roadmaps.	Rapidly exploring random graph (RRG) algorithm aids roadmap construction in maximum reachable insertion workspace.	Tip error between 0.18 mm and 0.21 mm of tip width.
Killian et al. [139]	Multicopter collision avoidance by redirecting a drone onto a planned path; connects random nodes within a search space on a virtual line.	Use of the probabilistic RRT algorithm for collision detection.	Speed of up to 6 m/s.

## Appendix A. State of the Art and Proof of Concept in AR-Based Surgical Robots from Existing Literature

**Author/** **Company**	**Name of** **Device**	**Parameters Studied**	**AR Interface**	**Type of** **AR Display**	**Operating Principle**	**Surgical** **Specialization**	**CE Marking**
Mazor Robotics Inc., Caesarea, Israel	SpineAssist [172]	CT-scan-based image reconstruction, path planning of screw placement, and needle tracking.	Graphical user interface for fluoroscopy guidance using fiducial markers.	Marker-based	The system is fixed to the spine, attached to a frame triangulated by percutaneously placed guidewires.	Transpedicular screw placement (orthopedic) Brain surgery	Yes (2011)
	Renaissance [173]	3D reconstruction of spine with selection of desired vertebral segments.	Hologram generation for localization of screw placement.	Superposition-based	Ten-times faster software processing for target localization due to DL algorithms.	Thoracolumbar screw placement (orthopedic)	Yes (2011)
Zimmer Biomet, Warsaw, Indiana	ROSA Spine [174]	Image reconstruction, path planning of screw placement, and needle tracking.	3D intraoperative planning software for robotic arm control.	Superposition-based	Robotic arm with floor-flexible base, which can readjust its orientation.	Transpedicular screw placement (orthopedic) Brain surgery	Yes (2015)
MedRobotics, Raynham, MA, USA	MazorX [175]	Image reconstruction, 3D volumetric assay of the surgical field.	3D intraoperative planning software for robotic arm control and execution.	Superposition-based	Matching preoperative and intraoperative fluoroscopy to reconstruct inner anatomy.	General spine and brain surgery	Yes (2017)
	Flex Robotic System [176]	Intraoperative visualization to give surgeons a clear view of the area of interest.	Built-in AR software with magnified HD for viewing of anatomy.	Superposition-based	Can navigate around paths at 180 degrees to reach deeper areas of interest in the body by a steering instrument, i.e., joystick. Use of two working channels.	Transoral robotic surgery (TORS), transoral laser microsurgery (TLM), and Flex^®^ procedures	Yes (2014)
Novarad^®^, Pasig, Philippines	VisAR [5]	Instrument tracking and navigation guidance, submillimeter accuracy.	Reconstructs patient imaging data into 3D holograms superimposed onto patient.	Superposition-based	Hands-free voice recognition for facilitated robot control. Voice User Interface (VUI). Automatic data uploading to the system.	Neurosurgery	Yes (May 2022)
Medacta, Castel San Pietro, Switzerland	NextAR [177]	Instrument tracking and 3D navigation guidance, submillimeter accuracy.	Use of smart glasses to deliver an immersive experience to surgeons.	Superposition/marker-based	Overlays 3D reconstructed models adapted to the patient’s anatomy and biomechanics.	CT-based knee ligament balance and other hip, shoulder, and joint arthroplasty interventions.	Yes (2021)
IMRIS Inc., Winnipeg, MB, Canada	NeuroARM [178]	MRI-based image-guided navigation, force feedback from controllers for tumor localization and resection.	AR-based immersive environment for recreation of haptic, olfactory, and touch stimuli.	Marker-based	Image-guided robotic interventions inside an MRI, with sensory stimulus from workstation to guide the end-effector.	Brain surgery	Yes (2016)
Ma et al., Chinese University of Hong Kong	6-DoF robotic stereo flexible endoscope (RSFE) [179]	Denavit–Hartenberg derivations of Jacobian, servo control, and head tracking for wider angle view, user evaluation, task load comparison.	HoloLens-based tracking using HMD for image-guided endoscopic tracking.	Marker-based	Use of head tracking HoloLens for camera calibration and visualization of tool placement of flexible endoscope	Cardiothoracic	No
Fotouhi et al., John Hopkins University	KUKA robot-based reflective AR [125]	User evaluation, camera-to-joint reference frame Euclidean distance compared for no AR, reflective mirror AR, and single-view AR, joint error calculation.	HMD-based robotic arm guidance and positioning using reflective mirrors.	Marker-based	Digital twin with ghost robot for mapping of virtual-to-real robot linkages from a reference point.	Cardiothoracic	No
Forte et al., Max Planck Institute for Intelligent Systems	Robotic dry-lab lymphadenectomy [180]	Distance computation for Euclidean arm measurements, user evaluation of AR alignment accuracy.	Stereo-view capture of medical images acquired by robot and HD visualization.	Marker-based	AR-based HMD used to visualize the motion of surgical tip in an image-guided procedure. Image processing of CT scans to locate pixels of virtual marker placed in virtual scene.	Custom laparoscopic box trainer containing a piece of simulated tissue	No
Qian et al., John Hopkins University	Augmented reality assistance for minimally invasive surgery [181]	Point cloud generation for localization of markers, system evaluation using accuracy parameters such as frame rate, peg transfer experiment.	Overlay of point clouds on test anatomy.	Superposition/rigid marker-based	AR-based experimental setup for guiding of a surgical tool to a defect in anatomy.	General surgery	No

## Appendix B. Types of Neural Networks Used in Image Registration for AR Reconstruction in Surgery

**Authors**	**Model**	**Performance Metrics**	**Purpose**	**Accuracy**	**Optimization Algorithm**	**Equipment**
Von Atzigen et al. [80]	Stereo neural networks (adapted from YOLO)	Bending parameters such as axial displacement, reorientation, bending time, frame rate.	Markerless navigation and localization of pedicles of screw heads.	67.26% to 76.51%	Perspective-n-point algorithm and random sample consensus (RANSAC), SLAM.	Head-mounted AR device (HoloLens) with C++
Doughty et al. [182]	SurgeonAssistNet composed of EfficientNet-Lite-B0 for feature extraction and gated recurrent unit RNN	Parameters of the GRU cell and dense layer, model size, inference time, accuracy, precision, and recall.	Evaluating the online performance of the HoloLens during virtual augmentation of anatomical landmarks.	5.2× decrease in CPU inference time.	7.4× fewer model parameters, achieved 10.2× faster FLOPS, and used 3× less time for inference with respect to SV-RCNet.	Optical see-through head-mounted displays
Tanzi et al. [118]	CNN-based architectures such as UNet, ResNet, MobileNet for semantic segmentation of data	Intersection over union (IoU), Euclidean distance between points of interest, geodesic distance, number of iterations per second (it/s).	Semantic segmentation of intraoperative proctectomy, for 3D reconstruction of virtual models to preserve nerves of the prostate.	IoU = 0.894 (σ = 0.076) compared to 0.339 (σ = 0.195).	CNN with encoder–decoder structure for real-time image segmentation and training of a dataset in Keras and TensorFlow.	In vivo robot-assisted radical prostatectomy using DaVinci surgical console
Brunet et al. [183]	Adapted UNet architecture for simulation of preoperative organs	Image registration frequency, latency between data acquisition, input displacements, stochastic gradients, target registration error (TRE).	Use of an artificial neural network to learn and predict mesh deformation in human anatomical boundaries.	Mean target registration error = 2.9 mm, 100× faster.	Immersed boundary methods (FEM, MJED, Multiplicative Jacobian Energy Decomposition) for discretization of non-linear material on mesh.	RGB-D cameras
Marahrens et al. [184]	Visual deep learning algorithm such as UNet, DC-Net	For autonomous robotic ultrasound using deep-learning-based control, for better kinematic sensing and orientation of the US probe with respect to the organ surface.	Semantic segmentation of vessel scans for organ deformation analysis using a dVRK and Philips L15-7io probe.	Final model Dice score of 0.887 as compared to 0.982 in [179].	DC-Net with images in the propagation direction feed through, binary classification task, IMU-fused kinematics for trajectory comparison.	Philips L15-i07 probe driven by US machine, dVRK software
